# Came True

**Published:** 1888-03

**Authors:** 


### CAME TRUE.

The Cannon Ball train from the east on the Texas and Pacific Railroad was running over the trestle in Bois Darc bottom recently, when the rails spread and caused the engine, baggage car, and one coach to leave the track. Fortunately the cars did not fall off the trestle, and no one was seriously hurt. The regular engineer, Dave Lasler, on being called at Texarkana to take charge of his engine, flatly refused to go out giving as his excuse that he had dreamed that the Cannon Ball would be thrown from the track that night between Texarkana and Bonham. Another engineer was procured.

